# Assessment of Capsular Contracture Based on Morphological Change of Breast Implant Using Computed Tomography

**DOI:** 10.1055/a-2620-3350

**Published:** 2025-07-23

**Authors:** Won Seok Oh, Seung Hoon Lee, Jae Woo Lee, Jung Yeol Seo, Choong Rak Kim, Su Bong Nam

**Affiliations:** 1 Department of Plastic and Reconstructive Surgery, Pusan National University School of Medicine, Yangsan, Korea (the Republic of); 2 Department of Statistics, Pusan National University, Busan, Korea (the Republic of)

**Keywords:** breast implant, capsular contracture, Computed tomography

## Abstract

**Background:**

Capsular contracture is a common complication following implant-based breast reconstruction. Current assessment methods, primarily relying on the subjective Baker grading system, lack objectivity and quantitative data, which hinders large-scale studies and the development of treatment guidelines. To solve these problems, we conducted a study using computed tomography (CT) scans to quantitatively evaluate morphological changes in breast implants associated with capsular contracture.

**Methods:**

We enrolled 94 patients who underwent breast reconstruction using implants and underwent periodic chest CT scans. We categorized them into two groups: Baker grade I or II ( *n* = 72) and Baker grade III or IV ( *n* = 22). We analyzed the CT scans to assess changes in the implant base and projection.

**Results:**

In the Baker grade III or IV groups, it was confirmed that the ratio of projection to base increased after capsular contracture compared with before contracture. On the other hand, there was no significant change in the ratio of projection to base in the Baker grade I or II groups.

**Conclusion:**

This study highlights the potential of CT scans as a reproducible method for evaluating capsular contracture. The ratio of projection to base could serve as a new quantitative index alongside the Baker grades for clinical assessment, treatment planning, and research on capsular contracture. When comparing the ratio of projection to base before and after capsular contracture, if the ratio of projection to base increases by more than 1.233 times, it can be considered Baker grade III or IV.

## Introduction

Capsular contracture is a common complication of breast augmentation and reconstruction using implants. 1 2 3 4 5 6 As the progression of capsular contracture may adversely affect aesthetic outcomes and cause pain, capsular contractures sometimes require surgical intervention.

The Baker grading system is most commonly used to evaluate capsular contracture 7 8 ; however, this system is subjective and does not provide quantitative data, which limits its use in large-scale studies and treatment guidelines. Consequently, there has been a growing need and attempt to develop more objective and quantitative evaluation methods for capsular contracture, but well-established techniques and ideas are lacking.

Previous studies have attempted to evaluate capsular contracture using imaging techniques, including ultrasound and magnetic resonance imaging (MRI), by assessing capsule thickness and implant surface characteristics. 9 10 11 12 13 14 15 However, these examinations are operator-dependent, and there is a lack of standardized indications for imaging studies. Additionally, there are controversies regarding the variability of capsule thickness based on signal intensity, and these techniques have low reproducibility across different health care facilities.

To establish objective diagnostic criteria, we quantitatively evaluated changes in breast implant morphology based on their projection and base according to the onset of capsular contracture and analyzed the statistical significance.

Computed tomography (CT) scans are better than MRI for analyzing breast shape because they are performed in the supine position. Additionally, breast cancer patients often undergo periodic CT scans to check for lung metastasis after surgery. Therefore, we quantitatively evaluated capsular contracture by analyzing CT images taken before and after capsular contracture.

The objective of this study was to quantitatively assess the morphological changes of breast implants following capsular contracture using CT and to conduct a feasibility study to identify imaging features associated with capsular contracture. Furthermore, we aim to propose the possibility of a new capsular contracture grading system to quantitatively evaluate breast implants and the surrounding pocket on CT scans.

## Methods

We enrolled 251 patients who underwent breast reconstruction using implants between May 2012 and February 2019. Patients who underwent nipple-sparing mastectomy or skin-sparing mastectomy along with breast reconstruction using latissimus dorsi (LD) implants, two-stage reconstruction, or direct-to-implant (DTI) reconstruction were included. All surgeries were performed by a single plastic surgeon. The surgeon scored capsular contracture using the Baker grade scoring system during outpatient follow-ups.

**Fig. 1:**
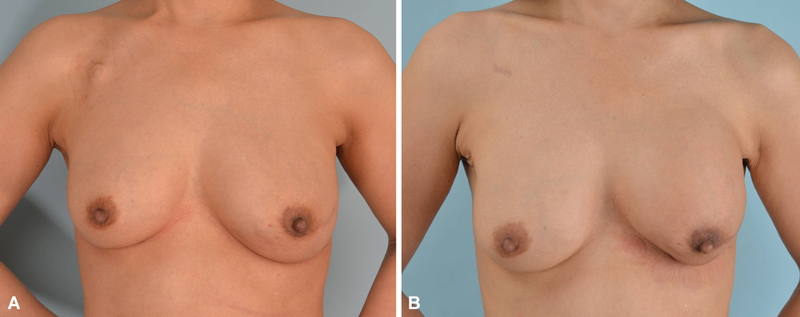
( **A** ) Before capsular contracture of the left breast, 6 months after left breast reconstruction using an implant. ( **B** ) After capsular contracture of the left breast (same patient as [A]), 28 months after left breast reconstruction using an implant.

We excluded 42 patients who developed complications other than capsular contracture and 92 patients who did not undergo periodic chest CT after implant insertion surgery. Also, 52 patients whose overall shape of the implants was not visible on chest CT and 12 patients who received anatomical implants were excluded. For four patients who underwent bilateral breast reconstruction, one side was randomly selected for the evaluation ( Fig. 1 ).

**Fig. 2:**
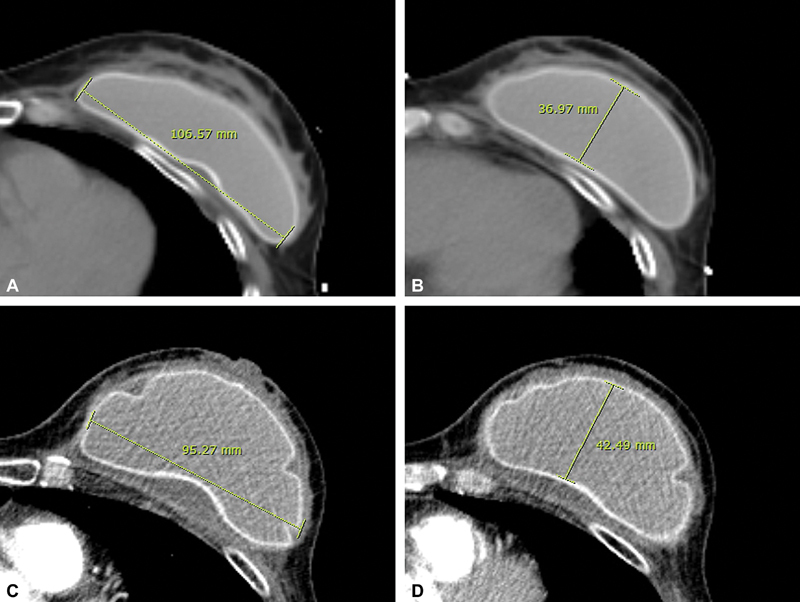
( **A** ) Base of implant before capsular contracture of the left breast (same patient as in Fig. 1 ). ( **B** ) Projection of implant before capsular contracture of the left breast (same patient as in Fig. 1 ). ( **C** ) Base of implant after capsular contracture of the left breast (same patient as in Fig. 1 ). ( **D** ) Projection of implant after capsular contracture of the left breast (same patient as in Fig. 1 ). The base length and perpendicular projection length of the implant in all slices where the implant was visible were measured and the highest values were selected on transverse CT scans. CT, computed tomography.

For Baker grade III or IV patients, we included those who had undergone chest CT before the onset of capsular contracture (2–18 months after surgery, average 6.94 months) and after the onset of capsular contracture (17–85 months after surgery, average 57.10 months). For Baker grade I or II patients, we enrolled those with at least one chest CT scan taken 2 to 18 months after surgery (average 7.19 months) and 23 to 79 months after surgery (average 49.54 months; Fig. 2 ).

Finally, 94 patients (94 breasts) were selected for analysis, comprising 72 Baker grade I or II patients and 22 Baker grade III or IV patients. For Baker grade I or II patients, measurements of the base and projection of the implant were taken from transverse views of chest CT scans performed at 2 to 18 months after surgery (average 7.19 months), and chest CT scans taken at 23 to 79 months after surgery (average 49.54 months). For Baker grade III or IV patients, measurements of the base and projection of the implant were taken from transverse views of chest CT scans obtained before the onset of capsular contracture (2–18 months after surgery, average 6.94 months) and after the onset of capsular contracture (17–85 months after surgery, average 57.10 months). Three plastic surgeons measured the base length and perpendicular projection length of the implant in all slices where the implant was visible and selected the highest values for both the base and projection. Then, the final values of projection and base were calculated by averaging the projection and base values obtained by three doctors. The ratio of projection to base was subsequently calculated, and an independent samples ***t*** -test was performed at a significance level of 0.001 to determine if there was a significant difference between the two groups. All the statistical computations are done by the statistical package R (version 4.3.1). 16

## Results

There were no significant differences in the mean age, body mass index (BMI), or implant type between the two groups. However, the proportion of patients who underwent postoperative radiotherapy (RTx) was significantly higher in the Baker grade III or IV group ( *p* < 0.001, Table 1 ).

**Table 1:** Patient demographics

Characteristics	Baker grades I, II ( *n* = 72)	Baker grades III, IV ( *n* = 22)	*p-* Value
Age, years	46.06	48.45	0.277
BMI, kg/m ^2^	23.06	22.71	0.697
Postoperative RTx	4	15	<0.001
Type of surgery			0.407
DTI	23	7	–
Two-staged	19	3	–
LD + implant	30	12	–
Implant size			0.247
Size <150 mL	14	6	–
150 mL ≤ Size <250 mL	30	6	–
250 mL ≤ Size <350 mL	18	9	–
350 mL ≤ Size	10	1	–

Abbreviation: BMI, body mass index; DTI, direct-to-implant; LD, latissimus dorsi; RTx, radiotherapy.

Among 22 patients in the Baker grade III or IV group, the mean base and projection measurements on chest CT were 105.364 mm and 32.500 mm, respectively, prior to capsular contracture (2–17 months after surgery, mean 6.94 months). After the onset of capsular contracture, the mean base and projection measurements on chest CT were 98.227 mm and 39.500 mm, respectively (17–85 months after surgery, mean 50.82 months). After the onset of capsular contracture, the base decreased by an average of 7.137 mm (6.77%, *p* < 0.001, Table 2 ), and the projection increased by an average of 7 mm (21.54%, *p* < 0.001, Table 3 ). In the Baker grade I or II group, the mean base and projection measurements on chest CT were 108.625 mm and 33.528 mm, respectively, at 2 to 25 months after surgery (mean 7.19 months). At 23 to 79 months (mean 49.54 months), the mean measurements were 109.403 mm and 33.681 mm, respectively. Base increased by an average of 0.777 mm ( *p* = 0.071, Table 2 ), and projection increased by an average of 0.153 mm (0.456%, *p* = 0.660, Table 3 ).

**Table 2:** Changes in implant base by Baker grades

Category		Descriptive statistics			*t* ( *p* )
		*N*	Mean (M)	Standard deviation (SD)	
Baker grades I, II	2–18 months after surgery	72	108.625	14.582	0.071
	23–79 months after surgery	72	109.403	14.137	
Baker grades III, IV	2–18 months after surgery	22	105.364	12.234	<0.001
	17–85 months after surgery	22	98.227	11.305	

**Table 3:** Changes in implant projection by Baker grades

Category		Descriptive statistics			*t* ( *p* )
		*N*	Mean (M)	Standard deviation (SD)	
Baker grades I, II	2–18 months after surgery	72	33.528	7.451	0.660
	23–79 months after surgery	72	33.681	7.805	
Baker grades III, IV	2–18 months after surgery	22	32.500	7.999	<0.001
	17–85 months after surgery	22	39.500	9.329	

We calculated the ratio of projection to base both before and after the onset of capsular contracture. In the Baker grade III or IV group, the ratio of projection to base was 0.3055 before contracture and 0.4009 after contracture, with an average rate of increase of 1.3178. On the other hand, in the Baker grade I or II group, the average increase in the ratio of projection to base is 1.0004. At a significance level of 0.001, the independent samples *t* -test reveals a statistically significant difference in means between the two groups. Therefore, the increase in the ratio of projection to base was significantly higher in patients with the Baker grade III or IV compared with those with the Baker grade I or II ( *p <* 0.001, Table 4 ).

**Table 4:** Changes in the ratio of projection to base by Baker grades

Category		Long-/short-term follow-up ratio			*t* ( *p* )
		*N*	Mean (M)	Standard deviation (SD)	
Baker grades	I, II	72	1.0004	0.0989	<0.001
	III, IV	22	1.3178	0.1735	

**Fig. 3:**
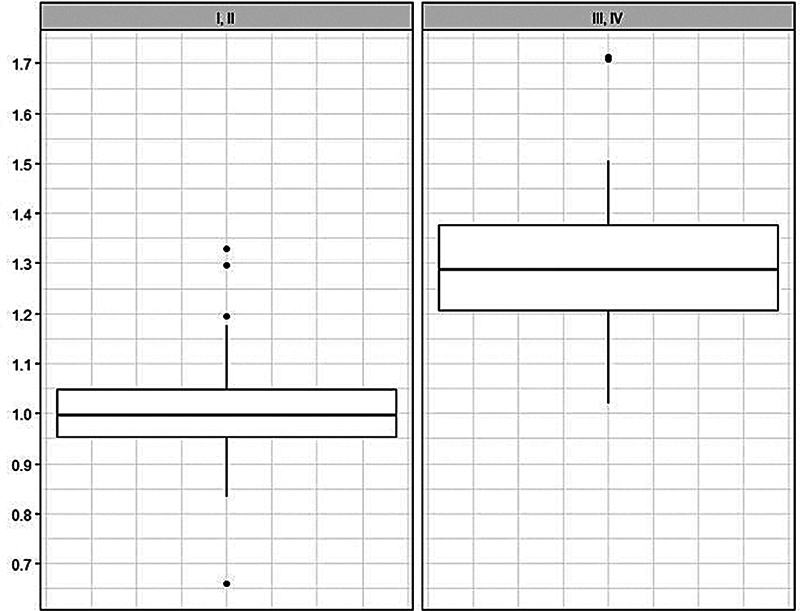
Box plot of changes in the ratio of projection to base by Baker grades.

**Fig. 4:**
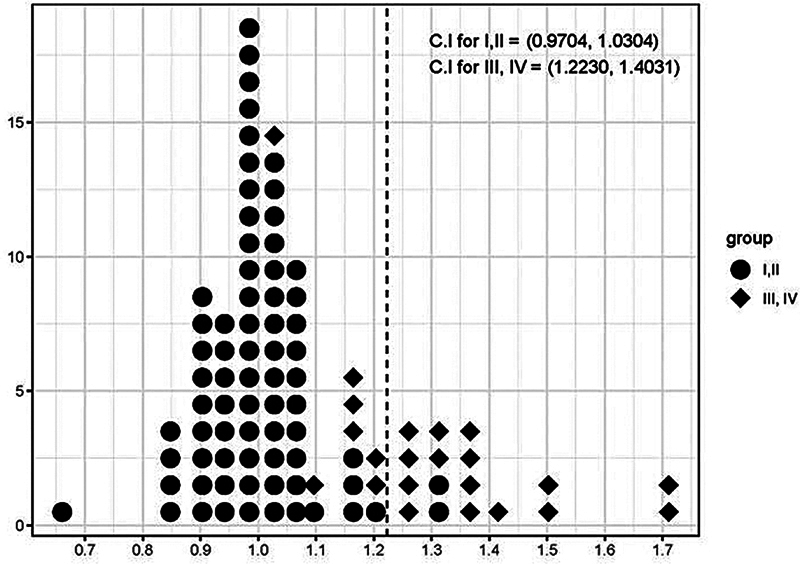
Dot plot of changes in the ratio of projection to base by Baker grades.

The 99% confidence interval for the increase rate of the ratio of projection to base in the Baker grade I or II is (0.9704, 1.0304), and in the Baker grade III or IV is (1.223, 1.4031) ( Figs. 3 and 4 ).

## Discussion

Capsular contracture is the most common complication and cause of patient dissatisfaction following breast augmentation or breast reconstruction using implants 1 ; previous studies have reported incidence rates of approximately 10%. 2 3 4 5 6 Progression of capsular contracture manifests diversely, including distorted breast shape and volume, increased firmness, and pain. Previous research has identified a variety of factors that contribute to capsular contracture, including breast augmentation or breast reconstruction, the texture of the implant surface, implant placement, infection, and bleeding. 7

Capsular contracture arises from abnormal responses of inflammatory cells, the extracellular matrix, and fibroblasts within the capsule formed around the implant. Fibroblasts, which facilitate wound contracture during healing and are lost, have been found to exhibit excessive and sustained responses in hypertrophic scarring or keloid formation. Similarly, it is believed that the excessive activation of fibroblasts within the capsule contributes to capsular contracture. 17 18

Currently, there is no universally accepted objective scale for capsular contracture. Various classifications have been proposed to score the severity of capsular contracture, but the Baker grading system remains the most widely used. 7 8 However, the Baker grading system relies on subjective assessment by both the patient and the physician, which limits its use as a standardized treatment criterion in hospitals and objective index in research. Additionally, it is possible that less experienced physicians may misdiagnose or miss the diagnosis of capsular contracture when using this system. As such, objective medical imaging techniques are essential to evaluate the severity of capsular contracture in clinical diagnosis and scientific research.

Many clinical studies have previously highlighted the importance of imaging techniques, including breast contrast studies, ultrasound, and MRI, for assessing the severity of capsular contracture. 13 14 15 19 In most studies, capsule thickness and morphological characteristics shown in the images have been utilized to assess the integrity of breast implants, and studies on capsular contracture have been predominantly limited to the surface morphology of implants and capsules. 9 10 11 12 20 Among the variety of available imaging techniques, MRI remains controversial, owing to the lack of standardized indications for MRI due to time and cost constraints and the low correlation between measured capsule thickness and capsular contracture. Although studies have attempted to quantify the relationship between capsular contracture and the roundness, eccentricity, and ratio length of implants measured on MRI, clinical reproducibility was low due to possible distortions in implant morphology when patients adopt the prone position required for MRI, as well as the relatively complicated methods. 10 Several studies using ultrasound for assessment have also been published. 9 21 However, it may be difficult to visualize the posterior boundary of the implant and the chest wall, and the findings may vary across examiners, as the results of these imaging techniques are dependent on operator expertise. It may further be difficult to accurately measure capsule thickness with both imaging techniques, as signal intensity varies between imaging periods. Additionally, capsule thickness does not adequately reflect individual patient characteristics, which can vary based on factors such as race, age, and tissue type. Several studies have further attempted to examine the relationship between the shape of implant folds and capsular contracture, but implant folds have limited utility due to their variability and weak correlation.

Traditionally, ultrasound and MRI are recognized as being suitable for the diagnosis of capsular contracture and monitoring of complications, with CT merely used as an adjunct method. As a result, relatively few studies have utilized CT, although there have been attempts to determine silicone breast implant rupture or capsular contracture based on implant deformation, number of folds, and capsule thickness. 22 However, no study utilizing CT to measure the implant roundness or ratio length has yet been conducted. For these reasons, we focused on using physical parameters that could be used as objective indices to address these issues.

**Fig. 5:**
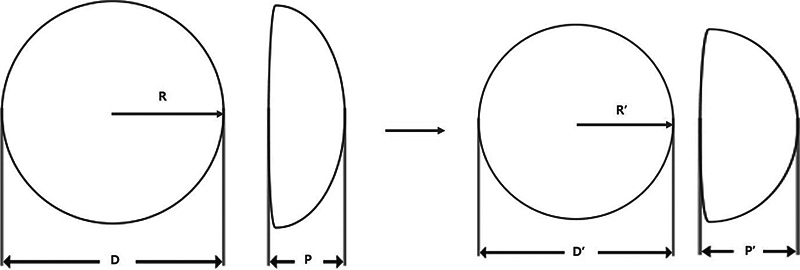
Anticipated changes in implant base and projection with capsular contracture.

We compared the CT images obtained immediately after surgery with those obtained after the onset of capsular contracture and observed a decrease in base and an increase in projection after the onset of capsular contracture. This led us to hypothesize that while the volume of the implant remains constant, the base would decrease while projection would increase as the implant becomes round due to changes in capsular tension, and we confirmed the statistical significance of these changes ( Fig. 5 ). Moreover, for cases graded Baker III or IV, the ratio of projection to base changed by 1.233 or higher after the onset of capsular contracture. Allowing quantitative evaluations of capsular contracture with high reproducibility in diverse clinical environments is a substantial clinical advantage for CT images.

This study has several limitations that should be mentioned. First, it was difficult to precisely determine the base and projection of the implant in both the transverse and sagittal views, as the thoracic cages of patients vary depending on whether or not the supine position was adopted during CT imaging. Converting CT images into 3D images to obtain more accurate implant base and projection measurements, and comparing them with the base implant specifications, would have produced more accurate results. However, as the aim of this study was to provide clinicians with an objective rate of change that could be easily used in practice, we tried to keep the measurement method as simple as possible. Thus, we decided to only use the ratio of projection to base under the assumption that patient-specific chest anatomy and positions would remain unchanged, and that imaging conditions would be the same before and after capsular contracture. Although the measurements could differ from the actual projection and base of the implant in the coronal or sagittal views, other variables could be controlled for if the same type of CT is performed. Additionally, chest CT scans are not universally required for breast reconstruction patients and are rarely indicated for cosmetic patients. Therefore, these findings may not be universally applicable.

Our findings suggest that it could be used to evaluate capsular contracture in clinical environments with high reproducibility. Furthermore, 3D CT images could promote additional research in this field. As performed in our study, CT could be used for quantitative assessments of capsular contracture based on simple measurements and equations in other health care facilities with different environments.

However, it is not necessary to use CT scans for diagnosing capsular contracture. Diagnosing capsular contracture should ideally be done through existing diagnostic criteria, and if there is a CT scan taken for other reasons, it is recommended to only use such indicators as references. Ultimately, diagnosing capsular contracture should rely on clinical correlation.

### Conclusion

Based on chest CT findings, it was confirmed that when capsular contracture appeared, the implant projection increased and the base decreased compared with immediately after surgery. If the ratio of projection to base increases by more than 1.233 times during the period when contracture is suspected, compared with immediately after surgery, it may indicate the possibility of capsular contracture belonging to the Baker grade III or IV group. Thus, we propose that the ratio of projection to base can potentially be used as a new quantitative index along with the Baker grades for clinical progress monitoring, treatment planning, and research regarding capsular contracture.
